# The Impact of Hybrid Compositional Film/Structure on Organic–Inorganic Perovskite Solar Cells

**DOI:** 10.3390/nano8060356

**Published:** 2018-05-23

**Authors:** Yinghui Wu, Wei Chen, Guo Chen, Liyu Liu, Zhubing He, Ruchuan Liu

**Affiliations:** 1 Department of Physics, Chongqing University, No. 55, University City South Rd., Chongqing 401331, China; yinghui@cqu.edu.cn (Y.W.); wezer@cqu.edu.cn (G.C.); lyliu@cqu.edu.cn (L.L.); 2 Department of Materials Science and Engineering, Shenzhen Key Laboratory of Full Spectral Solar Electricity Generation (FSSEG), Southern University of Science and Technology, No. 1088, Xueyuan Rd., Shenzhen 518055, China; chenw7@mail.sustc.edu.cn (W.C.); hezb@sustc.edu.cn (Z.H.)

**Keywords:** Cs-based perovskite, 2 dimensional, polymer, layered structure, bulk heterojunctions, composite-structured, perovskite solar cells

## Abstract

Perovskite solar cells (PSCs) have been intensively investigated over the last several years. Unprecedented progress has been made in improving their power conversion efficiency; however, the stability of perovskite materials and devices remains a major obstacle for the future commercialization of PSCs. In this review, recent progress in PSCs is summarized in terms of the hybridization of compositions and device architectures for PSCs, with special attention paid to device stability. A brief history of the development of PSCs is given, and their chemical structures, optoelectronic properties, and the different types of device architectures are discussed. Then, perovskite composition engineering is reviewed in detail, with particular emphasis on the cationic components and their impact on film morphology, the optoelectronic properties, device performance, and stability. In addition, the impact of two-dimensional and/or one-dimensional and nanostructured perovskites on structural and device stability is surveyed. Finally, a future outlook is proposed for potential resolutions to overcome the current issues.

## 1. Introduction

**Figure 1:**
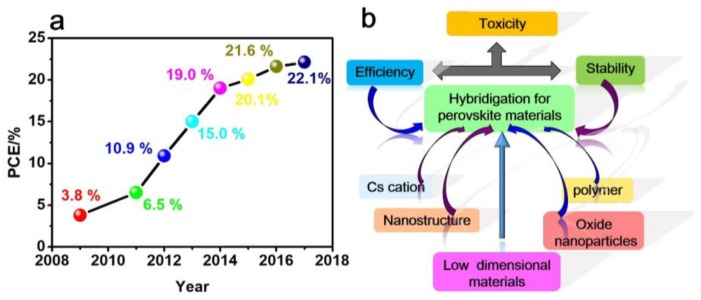
Introduction of (**a**) power conversion efficiency of perovskite solar cells over the years [4,5,6,7,8,9,10,11]; (**b**) the structure of the hybrid perovskite solar cells.

Perovskite solar cells (PSCs) have attracted immense research attention. They show unique properties such as a direct band gap, high absorption coefficient, and long carrier diffusion lengths [1,2]. The physical properties of the hybrid perovskites that are used in photo voltaic devices have been extensively studied, and are reviewed by Huang et al. in ref. [3]. Since the first report on dye-sensitized solar cells using a perovskite as the dye with an efficiency of 3.8% was published in 2009 [4], there has been a rapid increase in the number of publications in this area, as well as a rapid increase in the reported efficiencies. The evolution of the highest power conversion efficiencie (*PCE*) reported for PSCs over the years is depicted in Figure 1a [4,5,6,7,8,9,10,11]. A *PCE* of 22.1% was recently reached by Yang et al. [5]. Up to now, the highest reported efficiency of a perovskite solar cell was recorded at 22.7% by the National Renewable Energy Laboratory (NREL) [12]. The material used was an inorganic-organic hybrid material with a perovskite (ABX_3_) structure (A: methylammonium (CH_3_NH_3_^+^, MA), formamidinium (HC(NH_2_)_2_^+^, FA), Cs, Rb; B = Pb, Sn; X = halogen anion (Cl, Br and I), (SCN^−^)) [13,14,15,16,17]. Despite rapid developments in the performance of perovskite solar cells, there have been concerns about several issues such as the photocurrent hysteresis, device stability, and scaling issues that are able to affect the measurement accuracy and/or practical applications of these devices. In addition, there are possible environmental effects related to the use of lead-based perovskite materials [18,19,20].

In order to overcome the issues mentioned above, researchers have developed various strategies, such as hybrid components and nanostructures (Figure 1b). In this review, we briefly discuss the development of two main strategies for improving the stability of PSCs. Firstly, we will discuss hybrid materials combining alkali cations (i.e., Cs^+^) with organic cations. The incorporation of Cs^+^ can reduce the defect density and charge recombination rate, and enhance the ultraviolet and moisture resistance, thus improving the stability of PSCs [5,19,21,22,23]. Secondly, the development of multidimensional (3-D/2-D/1-D) perovskites to improve their structural stability will be discussed. A two-dimensional (2-D) perovskite film has higher stability and superior and controllable exciton properties, but the intense excitons greatly weaken its photoelectric properties [24,25]. Pure 2-D perovskite-based PSCs usually have low efficiencies due to the large insulating organic cations weakening the charge transport [26]. Using composites of 2-D layered perovskites and three-dimensional (3-D) hybrid perovskites is an effective strategy to improve device stability while maintaining high efficiency [27,28,29,30,31,32,33]. In addition, this review will also summarize the progress made with hybrid solar cells using polymers, metal oxides, Cs cation, and nanostructured perovskites, and the impact of these materials on PSCs’ efficiency and stability [34,35,36,37]. Finally, future developments that may solve the stability issues are presented.

## 2. Organic–Inorganic Hybrid Perovskites

In this section, we will discuss the properties of perovskites, including their physical properties and the effects of mixed ions.

### 2.1. Chemical Structures of Perovskites

**Figure 2:**
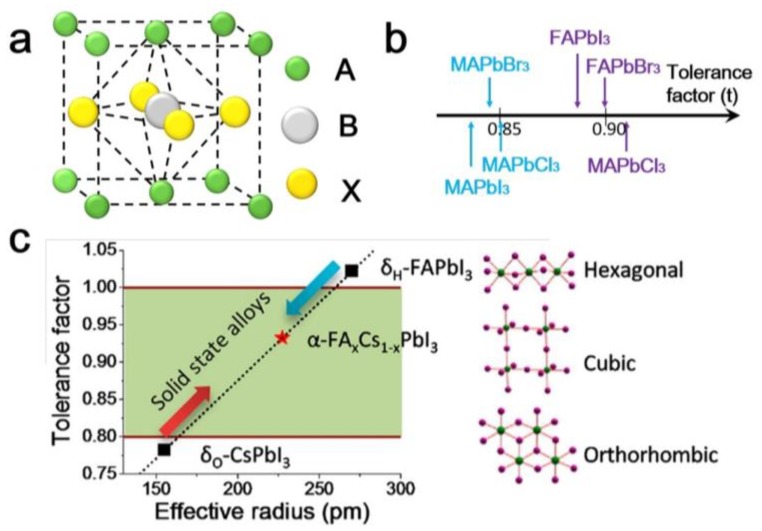
Physical properties of perovskites. (**a**) Schematic illustration of an ideal perovskite structure. The correlation between the Goldschmidt tolerance factor and the perovskite structure of (**b**) a typical perovskite and (**c**) Cs-FA cation perovskite. Reprinted with permission from ref. [41] Copyright 2016 American Chemical Society.

In general, the structure of a perovskite is ABX_3_. The name derives from CaTiO_3_, which was first reported in the 1920s by Goldschmidt et al. [38]. A and B are cations of different sizes, and X is the anion that balances the charges of both cations. The ideal structure of a perovskite is cubic. It is composed of a framework of corner-shared BX_6_ octahedra with 12 coordinated A cations, where the A-site cations sit on the corners of the cube, the B-site cation is located at the center of the octahedron (BX_6_), and the X ions are located on the surfaces of the cube, as shown in Figure 2a. Due to ion permutations and other factors, the crystal structure may be distorted. The structural transformations from orthorhombic to tetragonal and then to cubic perovskite take place at different temperatures [39]. The orthorhombic, tetragonal, and cubic structures are illustrated schematically in Figure 2c. The Goldschmidt tolerance factor is usually used to describe whether the crystals have an ABX_6_ structure [15,40]. In the perovskite structure, the ionic radii and the tolerance factor (*t*) follow the relationship: $t=\frac{R_{A}+R_{B}}{\sqrt{2}(R_{A}+R_{B})}$, where *R_A_*, *R_B_*, and *R_x_* are the ionic radii of the A-site cation, B-site cation, and X-site anion, respectively [41]. In an ideal structure, *t* is ~1. When 0.75 < *t* < 1.05, a distorted perovskite structure can normally be stabilized; when *t* < 0.75, the structure is that of ferrotitanium; and *t* > 1.1 is found in calcite or stone-type structures. The correlation between the perovskite structure and the tolerance factor is shown in Figure 2b,c [41,42]. To maintain the ABX_3_ structure in perovskites, it is necessary to match the radii of the A, B, and X ions.

### 2.2. Tuning Photoelectric Properties with Hybrid Cations and Anions in Perovskites

**Figure 3:**
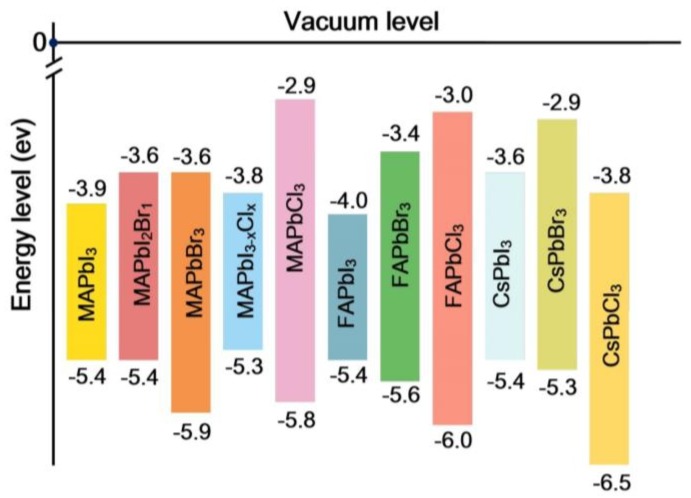
Energy-level diagram showing the valence band (VB) and conduction band (CB) levels of several typical perovskites [43,44,45,46,47,48].

The selection of the A-cation plays an important role in the regulation of the photoelectric properties of ABX_3_ perovskites. At present, the cations FA^+^, MA^+^, Cs^+^ and Rb^+^ are commonly used in perovskite structures. These cations can be ordered by size as follows: FA^+^ > MA^+^ > Cs^+^ > Rb^+^ [15]. The band gaps of some typical perovskites are shown in Figure 3 [43,44,45,46,47,48]. In theory, when the ionic radius increases, the cell expands, the forbidden band is narrowed, and the absorption spectrum is red-shifted. In contrast, when the ion radius decreases, the cell shrinks, the forbidden band is widened, and the absorption spectrum is blue-shifted.

Changing the size of the X anion can also tune the band gap of the perovskite. In the case of halogen anions, the lattice constant of ABX_3_ can be increased by substituting I for Cl, thus increasing the ionic radius, and the absorption spectrum of the perovskite film will be red-shifted [49]. Perovskites with mixed X anions have been reported in the literature, with studies of the average grain size, root mean square (RMS) roughness, grain boundaries, conductivity, and other properties [50]. The addition of Br and Cl can play a regulating role. Studies have shown that the addition of Cl promotes non-uniform nucleation at different positions, improves the morphology of the film, and thus facilitates carrier transport and reduces carrier recombination [10,51,52,53,54]. The addition of Br improves the charge transfer performance and device stability while enhancing the band gap [55,56,57,58]. Too many Br^−^ and Cl^−^ ions can increase the width of the forbidden band of the perovskite film, while decreasing the absorption and crystallinity. Reasonable regulation of the content of different halogen elements in perovskite materials can adjust their morphology and photoelectric properties. This method forms a stable cubic phase and inhibits non-radiative recombination by adjusting the A, B, and X ions. In the process of preparing different perovskite films, researchers have explored and optimized different combinations and proportions of cations and halides [59].

## 3. Solar Cells Based on Hybrid Perovskites

In the following section, we will discuss the properties of PSCs.

### 3.1. A Brief Summary of Key Parameters for Perovskite Solar Cells

**Figure 4:**
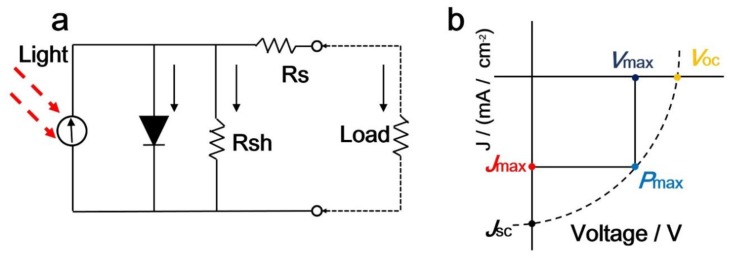
Solar cells can be described by an (**a**) equivalent circuit; (**b**) Example of a current density-voltage profile.

The detailed principles of solar cells have been explained by Nelson et al. in ref. [60]. In brief, the equivalent circuit diagram of a solar cell is displayed in Figure 4a. The equivalent circuit parameters are the series resistance, shunt resistance, and load [61]. When light is irradiating the device (Figure 4b), the voltage across the device is biased with the load. Therefore, the characteristics of perovskite solar cells are determined by measuring the current density. The *PCE* is evaluated from the short-circuit density (*J_sc_*) and the open-circuit voltage (*V_oc_*) using the following equation [62]: $PCE=\frac{J_{SC}V_{OC}}{R_{solar}}FF$, where *P*_max_ and *FF* are the incident power from the solar irradiation and fill factor, respectively. The value of the *FF* is the ratio of *P_max_* (*J*_max_*V*_max_) to the product of *J_sc_* and *V_oc_*. It should be noted that these important parameters have provided great guidance for evaluating PSCs [63].

The series resistance (*R_s_*) is real, and is defined as the sum of the resistances at the interface of each layer of the perovskite solar cell. The series resistance does not affect the open-circuit voltage of a PSC. However, it does affect the rate at which the voltage decreases with increasing current. For instance, a higher series resistance leads to a more rapid decrease of output voltage with increasing current. In addition, a higher series resistance leads to a lower fill factor and a reduced short-circuit current density of the perovskite solar cell [15]. The shunt resistance (*R_sh_*) is a parameter that describes resistance due to the recombination of charge carriers. The shunt resistance mainly affects the rate of change of the current with voltage. A lower shunt resistance can cause the internal circuit current to increase, and the external circuit current to decrease. The shunt resistance has a limited impact on the short-circuit current density of perovskite solar cells, but a too small shunt resistance will affect the open-circuit voltage [15]. In general, it is necessary to reduce the series resistance and increase the shunt resistance to improve the performance of perovskite solar cells.

### 3.2. Hybrid Architectures in PSCs

**Figure 5:**
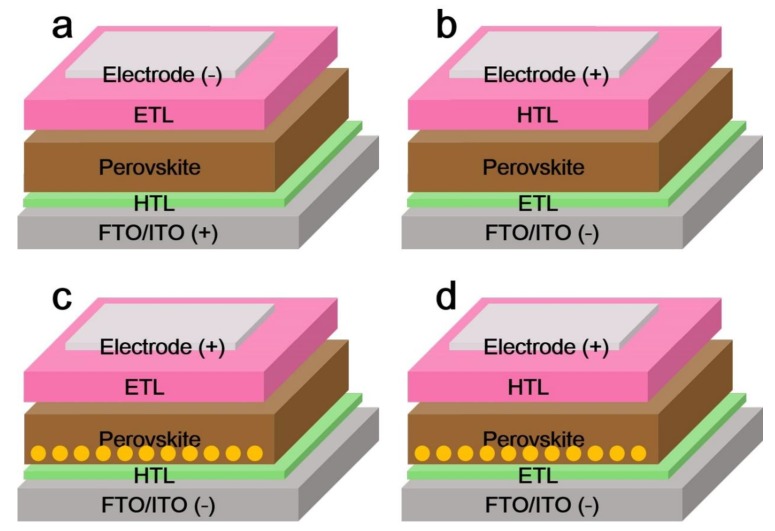
Structures of (**a**) inverted (p-i-n) and (**b**) normal planar structure (n-i-p) perovskite solar cells, (**c**) mesoporous structured (**c**) inverted (p-i-n) and (**d**) normal planar structure (n-i-p) perovskite solar cells. ETL and HTL represent the electron transport layer and hole transport layer, respectively.

Figure 5a,b shows the common architectures of perovskite solar cells, including p-i-n and n-i-p structures. PSCs can also be fabricated in two major types: planar and mesoporous structures, which are also shown in Figure 5. The architectures of p-i-n and n-i-p mesoporous structures are shown in Figure 5c,d. Guo et al. in 2013 designed the first p-i-n type perovskite solar cell consisting of an ITO/PEDOT:PSS/MAPbI_3_/PCBM/BCP/Al structure, and achieved a *PCE* of 3.9% [64]. Here, the poly(3,4-ethylenedioxythiophene) polystyrene sulfonate (PEDOT:PSS) served as a p-type layer and fullerene-based molecules served as the n-type layer to form the first p-i-n type perovskite solar cell. Poly(3,4-ethylenedioxythiophene) polystyrene sulfonate (PEDOT:PSS) [65,66,67] and transition metal oxides such as NiO*_x_* [68,69,70,71,72], CuO*_x_* [73,74,75], CrO*_x_* [76,77], and V_2_O_5_ [78,79], have been widely employed as hole-transporting materials in perovskite solar cells, demonstrating the importance of the hole-transporting layer (HTL) in PSCs. The HTL is usually spin-coated onto the fluorine-doped tin oxide (FTO) or tin-doped indium oxide (ITO) substrate before annealing, and serves to extract holes from the perovskite and then transfer them into the electrode. An n-type electron transporting layer (ETL), usually a metal oxide, such as TiO_2_ [8,80,81], SnO_2_ [21,82,83,84,85,86,87], or ZnO [88,89,90], can be used in PSCs. In addition, organic ETLs, such as [6,6]-phenyl-C_61_-butyric acid methyl ester (PCBM) [72,91], and C_60_ [92,93], have also been applied to PSCs. The ETL serves a similar function as the HTL, except that the charge carriers are electrons. In the above planar-layered architectures, the FTO or ITO and perovskite layers avoid direct contact. In contrast, in mesoporous PSCs, the mesoporous film serves not only as a transport layer, but also as a scaffold layer. Meanwhile, the creation of mesoporous oxide films, such as TiO_2_ [94,95,96], Al_2_O_3_ [10,97,98], and ZrO_2_ [99,100], have also been extensively investigated. A p-type hole-transporting material, such as polytriarylamine (PTAA) [101,102,103,104] or 2,2′,7,7′-tetrakis-(*N*,*N*-di-*p-*methoxyphenylamine)9,9′-spirobifluorene (spiro-OMeTAD) [105,106], is commonly used as the HTL in PSCs. The ETL/HTL must have a CB (conduction band)/VB (valence band) that is compatible with that of the perovskite that is used in planar/mesoporous perovskite solar cells. In consequence, the designs and material properties of the ETL/HTL are crucial for PSC performance.

## 4. Hybrid Cations in Perovskites for Improvements in PSC Stability

The significant degradation of organic perovskites after prolonged aging at high temperature and humidity continues to pose a challenge [107,108]. All-inorganic perovskites of the CsPbX_3_ form have attracted a lot of research attention due to their reduced volatility compared with organic hybrid halide perovskites. However, CsPbI_3_ devices have demonstrated poor efficiencies. This is because the black perovskite phase requires a high annealing temperature of over 300 °C in bulk [109,110], while at room temperature, a yellow orthorhombic phase with a wide band gap (1.73 eV) may exist [111]. This band gap is not suitable for high-efficiency solar cells. Therefore, the organic cation is an essential component in high-efficiency metal halide perovskite solar cells. As mentioned above, the different sizes of the A cation can expand or contract the lattice. Therefore, changing the size of the A cation can lead to changes in the B–X bond length in the ABX_3_ structure, which has been shown to influence the optical properties, i.e., the band gap [13,111]. In contrast to MA^+^, which has a large dipole moment, Cs^+^ cations have no dipole moment [112]. Both MA^+^ and FA^+^ cations have a larger tolerance factor for tuning the Cs-based perovskite system [41,42]. Therefore, through a combination of organic and inorganic cations with various ratios, hybrid cation-based perovskites show tremendous potential for improving the structural stability and manipulating the morphology and optoelectronic properties of perovskites, and thus enhancing the final device performance. In order to achieve better efficiency and stability, researchers have conducted extensive device optimization studies, focusing on the perovskite layer.

### 4.1. Cs-MA Hybrid Cation PSCs

The progress on Cs-MA cation perovskite systems is summarized in Table 1. Choi et al. reported the first Cs*_x_*MA_1−*x*_PbI_3_-based devices in 2014 [113]. The short-circuit current and open-circuit voltage of the perovskite/fullerene PSCs was improved by using the Cs-doped perovskite. Moreover, the absorption spectrum of the perovskite film could be changed by adjusting the doping ratio of Cs. Such an optimization of perovskite films not only improves the surface morphology, it also increases the band gap between the VB of the perovskite and the lowest unoccupied molecular orbital level of PCBM. Niemann et al. [114] and Niu et al. [115] improved the *PCE* by optimizing the optical and electronic properties with an appropriate composition ratio of Cs^+^ and MA^+^ cations. The introduction of Cs^+^ into the precursor solution by one-step spin-coating would inevitably accelerate the film deposition rate, leading to a smaller grain size. In addition to Cs^+^, Niu et al. [116] and Zhang et al. [117] also incorporated Br^−^ into the perovskite layer. In this way, Niu et al. were able to control the orientation ((112)/(200)) of the pure tetragonal phase of (MAPbI_3_)_1−*x*_(CsPbBr_3_)*_x_*. In this oriented film, the charge transfer was improved and trap states were suppressed, resulting in an enhanced *PCE*. Meanwhile, the incorporation of Cs^+^ and Br^−^ caused the unit cell to contract, also improving the resistance of the perovskite layer to moisture. Zhang et al. used CsBr for doping, and discovered the expansion of crystal grains and the enhancement of optical absorption due to the replacement of I^−^ by Br^−^ forming bonds with Pb^2+^. These results suggest that a better *PCE* can be achieved by hybrid perovskites doped with both Cs^+^ and Br^−^ ions, than with PSCs doped with ether Br^−^ or Cs^+^.

In addition to the traditional solution-processed method, other approaches to achieve the large-scale deposition of hybrid perovskite films have also been attempted. Chang et al. [118] applied the MA gas healing method to Cs-containing perovskite films. In this case, a certain amount of Cs cations were able to enter the crystal lattice and greatly enhanced the interactions between the inorganic framework and the more symmetrical PbI_6_ octahedra. Consequently, the humidity stability under continuous illumination was significantly improved. Sedighi et al. [119] utilized an atmospheric pressure vapor-assisted solution method to fabricate pinhole-free Cs*_x_*MA_1−*x*_PbI_3_ films. These Cs-doped films showed an increased open-circuit voltage due to their higher recombination resistance, and an enhanced optical absorption in the visible and near infrared region due to the increased grain size, as compared with MAPbI_3_ films.

All of these attempts to optimize Cs-doped perovskite films for PSCs suggest the importance of hybrid Cs^+^ cations. Cs doping can reduce the charge recombination process, and enhance the *V_oc_*, as well as improve the *PCE* and stability of PSCs.

### 4.2. Cs-FA Hybrid Cation PSCs

Cs-FA cation hybrid perovskites have also been extensively studied and optimized, and a brief summary is given in Table 2 [13,14,120,121,122]. In all of these studies, Cs-FA cation perovskite systems with Cs quantities from 0.1 to 0.2 displayed improvements in *PCE*, because the Cs-FA cation hybrid perovskites are more phase-stable. In order to obtain a better understanding, these hybrid perovskites have been examined via theoretical and experimental studies [123,124,125]. In particular, the CB minimum and VB maximum of Cs*_x_*FA_1−*x*_PbI_3_ have been evaluated [126].

Lee et al. found that the partial substitution of FA^+^ by Cs^+^ achieved better moisture stability than pure FAPbI_3_ perovskites [127], and an average *PCE* performance of 16.5% was realized. The high *PCE* was due to the suppression of charge recombination, which was attributed to the reduced trap density near the VB maximum. This led to an increase in the shunt resistance, open-circuit voltage, and fill factor. In the work by Li et al. [41], the tolerance factors of perovskite films with different Cs/FA ratios were estimated, suggesting that the tuning of the tolerance factor is a crucial step to stabilize the perovskite structure. This was achieved by the microstructure-mediated δ→α phase transformation, and the α phase was stabilized in the Cs-FA cation system at an annealing temperature lower than 150 °C. The best *PCE* of 17.2% was achieved with the hybrid Cs_0.15_FA_0.85_ perovskites in the α phase. In other articles, this phase transformation has been further studied in detail [121,124,128,135,136].

A better understanding of the phase transition in Cs-FA cation perovskite films can guide the optimization of PSC performance. There are various ways to control the phase of perovskites. Kim et al. reduced the defect density by an amide-catalyzed phase-selective crystallization process, thus enhancing the *PCE* of Cs-FA hybrid PSCs [130]. The optimized PSCs exhibited remarkably improved performance: a high *V_oc_* of 1.23 V, *J_sc_* of 18.34 mA·cm^−2^, an *FF* of 0.79, and a *PCE* of 17.8%. The high open-circuit voltage and stability of the PSCs were realized by controlling the film crystallization. Formamide (CH_3_NO) was introduced into the precursor solution as a highly polar additive, increasing the solubility of the Cs salt. In this way, the formation of the yellow phase was inhibited, and the ideal black phase was directly crystallized, reducing the defect density in the perovskite film. After 60 h of thermal stress, 85% of the initial film absorption was sustained. Among these perovskites, FA_0.83_Cs_0.17_Pb(I_0.5_Br_0.5_)_3_ showed the highest thermal resistance. The phase control of Cs-FA hybrid perovskites may also improve other factors in PSCs. Luo et al. fabricated Cs*_x_*FA_1−*x*_PbI_3_ films by Formamidinium iodide (FAI)gas phase-assisted compositional modulation, resulting in efficient and stable PSCs [132]. Wu et al. improved the carrier collection to enhance the *PCE* of Cs-FA double-cation PSCs by heterojunction engineering [120]. Conings et al. reported a DMSO-PbX_2_ “complex-assisted gas quenching” solution deposition method to fabricate pinhole-free perovskite layers [133]. Yu et al. prepared perovskite films with larger grains, resulting in enhanced carrier lifetimes, using an optimal 0.5 mol % Pb(SCN)_2_ additive, which made up for drawbacks caused by Cs substitutions (e.g., smaller grain sizes and shorter carrier lifetimes), and resulted in a noticeable improvement in fill factor [129]. The replacement of iodide with a limited amount of bromide was also reportedly used to obtain a high *PCE*, and the Cs*_x_*FA_1−*x*_Pb(I_1−*y*_Br*_y_*)_3_ film was found to possess a much higher stability due to the substitutions of Cs for FA, and Br for I [128].

At present, the solution spin-coating method is widely used to prepare perovskite thin films in the laboratory [102,137]. However, the *PCE* of PSCs made in this way decreases when the effective area of the PSCs is increased. Jiang et al. proposed a new method to solve this problem [126]. In their approach, FAPbI_3_ perovskite films were first prepared by a hybrid chemical vapor deposition process; then, the FAPbI_3_ was transformed into the more stable Cs*_x_*FA_1−*x*_PbI_3_ by a cation exchange method. The conversion process is very simple and fast, and can be completed in a few seconds. Among these Cs*_x_*FA_1−*x*_PbI_3_ films, the PSCs using Cs_0.07_FA_0.93_PbI_3_ showed the highest *PCE*, and accordingly, the device stability was significantly improved. The *PCE* of PSCs prepared by this method reached 14.6% with an effective area of 12.0 cm^2^. After 1200 min of continuous sun exposure and simultaneous load, the device performance showed no attenuation. Furthermore, while the effective cell area was dramatically increased, the *PCE* of the PSCs prepared in this way was not significantly decreased. Wide band gap (FA-Cs)Pb(I-Br)_3_ hybrid perovskites are promising materials for tandem solar cells. Great progress has been made in tandem solar cells in the form of perovskite-perovskite and perovskite-silicon tandem solar cells [125,131,138,139]. In such cells, the energy gap (Eg) of a Cs-FA hybrid perovskite is adjusted close to 1.75 eV, the optimal Eg of the top cell material, to match that of the bottom C–Si cell. Tandem solar cells can better absorb the full solar spectrum, making these PSCs closer to commercialization.

These results showed that the addition of Cs^+^ and Br^−^ is conducive to the stability of the cubic phase. It not only improves the photoelectric properties of the PSCs, but also enhances the charge extraction. In addition to the improved *PCE*, it also significantly improves the thermal stability and phase stability of the PSCs, providing new guidance for the design of perovskite materials.

### 4.3. Multication Hybridization for PSCs

Compared with the above binary hybridizations, Cs/FA/MA cation hybrid PSCs have also been attempted, showing greatly improved optical and electrical properties; accordingly, these devices have shown excellent reproducibility. Moreover, the band gap energy can be tuned in a wide range to meet the demands of a highly efficient solar cell. Theoretical investigations have demonstrated that these multication hybrids can greatly improve the stability and moisture resistance of PSCs [19,140,141,142,143].

Saliba et al. first reported Cs, FA, and MA triple-cation perovskites [144]. A stabilized power output of 21.1% with a *J_sc_* of 23.5 mA·cm^−2^, a *V_oc_* of 1.07 V, and a *FF* of 0.74 was achieved. After 250 h in a nitrogen atmosphere held at room temperature, the *PCE* remained at ~18%. A ratio of 5% CsI was found to be best when incorporated into the (FAPbI_3_)_0.83_(MAPbBr_3_)_0.17_ hybrid cation perovskite. The incorporation of Cs^+^ suppressed the non-perovskite phase and enhanced the crystallization process. Although the *PCE* can be improved by changing the chemical composition and the tunable band gap, the performance of PSCs is limited by non-radiative losses. In the continuously tunable band gap perovskite, photoinduced ion segregation leads to the instability of the band gap. For better optimization, Mojtaba et al. made a series of glass passivated triple-cationic perovskite films in 2018 [22]. They noticed that the standard triple-cationic precursor solution had a slight halogen defect, so they used a precursor solution diluted with potassium iodide. The introduction of potassium iodide led to a slight excess of halide in the sample and a very small change in the I/Br ratio. The resulting perovskite films consisted of uniformly filled grains, each of which was ~200–400 nm. The absorption and photoluminescence spectra showed that with the addition of potassium iodide, the optical band gap of the perovskite film decreased. The non-radiative dissipation and photoinduced ionic migration in the perovskite films and at the interfaces were greatly alleviated by using a passivated potassium halide layer at the surface and grain boundaries.

At the same time, the addition of Rb has also been attempted. It led to an enhanced charge carrier mobility, but little improvement in the defects in the perovskite layer [145]. The efficiency of the device was slightly increased and the current voltage hysteresis effect was reduced. In contrast, the incorporation of cesium can reduce the defect density and charge recombination rate. By combining both Cs^+^ and Rb^+^ cations, the researchers observed the highest level of photoelectric charge mobility and the lowest defect density, which gave the solar cells the highest stable output power. Therefore, the bottleneck limiting the *PCE* of PSCs is mainly the number and nature of the defect states rather than the carrier mobility of the perovskite layer.

The application of multiplecation (Cs/FA/MA/Rb) systems has become a good strategy for developing stable, high-efficiency perovskite solar cells. The reported performances of some high-efficiency PSCs are summarized in Table 3 in detail. These works demonstrate that the multication hybrid perovskite is an effective approach for preparing stable, high-efficiency PSCs at a low temperature. This also shows the great potential of the perovskite solar cell for commercial applications.

## 5. Hybrid-Dimension Perovskite-Based PSCs

The long-term stability issues preventing the use of 3-D perovskites in industrial applications are more outstanding in ambient air for the following reasons. First, lead in the perovskite can easily be oxidized and volatilized. Second, water in the air can easily break down perovskite materials. Third, ion migration and thermal decomposition also lead to instability. To overcome these issues, low-dimensional perovskite materials have been introduced by the replacement of MA/FA with large organic amines, including phenylethylammonium (PEA) [27,154,155], poly(ethyleneimine) (PEI) [156], butylamine (BA) [26,157], cyclopropylamine (CA) [31], and cyclohexylammonium iodide (CHMA) [158]. Low-dimensional structures are formed from alternating organic amine layers and inorganic layers. In consequence, the smaller the number of octahedral layers, the closer the perovskite is to a two-dimensional layer.

Compared with traditional 3-D perovskite structures, low-dimensional perovskite materials have certain advantages for PSCs. The introduction of large organic amines greatly enhances moisture resistance and photothermal stability. In addition, the modulation of optical and electrical properties can be realized by changing the type of the organic amine. However, low-dimensional perovskites have a large optical band gap, and the introduction of an organic amine reduces the carrier mobility. As a result, the measured efficiencies of low-dimensional PSCs have been significantly lower than those of the 3-D PSCs [24,25,26,159]. Therefore, combining the merits of both 3-D and low-dimensional perovskites has become an attractive research goal. In this way, enhancements in both efficiency and stability may be realized. Solar cells based on such dimensional hybrid perovskites with different-sized cations have been studied. In the following section, some facile methods for maintaining efficiency while solving the stability problem are reviewed.

### 5.1. Hybrid 1-D/2-D/3-D Structured PSCs

To improve stability, one-dimensional (1-D) and two-dimensional perovskites can be incorporated to the precursor solution and dispersed into a perovskite film (as seen in Table 4). The aim is to achieve highly efficient and stable PSCs by adjusting the morphology, grain size, band gap, carrier mobility, and other properties. In traditional 3-D perovskite films, the incorporation of a 2-D perovskite material is a reasonable strategy to achieve higher stability while maintaining the high efficiency.

Koh et al. used the immersion method to prepare dimensional hybrid PSCs, which were more than 9% efficient [160]. The two-dimensional perovskite thin films were immersed in a solution of organic small molecule amine (MAI). By adjusting the soaking time (1–5 min), various dimensional hybrid perovskites could be obtained. In addition, the films obtained by the immersion method still maintained a very good moisture resistance. Grancini et al. found a way to overcome the instability of PSCs by designing a 2-D/3-D (HOOC(CH_2_)_4_NH_3_)_2_PbI_4_/CH_3_NH_3_PbI_3_ perovskite junction [161]. In addition to the enhanced stability, these PSCs can effectively absorb light over the entire visible spectrum and transmit electric charge. The efficiencies of their PCS were 12.7% (carbon-based structure) and 14.6% (standard mesoporous solar cells), respectively. A *PCE* of 11.2% was realized using a fully printable process at the industrial scale. Device performance was maintained for more than 10,000 h under standard conditions. Wang et al. fabricated bulk heterojunctions by mixing a perovskite with the fullerene derivative A_10_C_60_ by solution-processing [162]. The composite was formed from an ethanol solution consisting of CH_3_NH_3_PbI_3_ and A_10_C_60_, with a large interfacial area between them. The goal here is to balance the charge extraction efficiencies by making up for the different diffusion lengths of holes and electrons, thus enhancing the *PCE*. Wang et al. produced PSCs with both high efficiency (20.6%) and stability by incorporating butylammonium in the Cs-FA cation perovskite to form 2-D/3-D heterostructures [163]. In this case, the 2-D perovskite was distributed among the highly oriented 3-D perovskite grains. This inhibited non-radiative charge recombination by reducing the hysteresis, enhancing the efficiency, and improving the stability. The relationship between composition, crystal orientation, and device performance was also investigated. The PSCs with optimal butylammonium content showed an average stable *PCE* of 17.5 ± 1.3% with a 1.61-eV band gap and a *PCE* of 15.8 ± 0.8% with a 1.72-eV band gap, respectively. The device stability under light was also improved. After 1000 h of illumination in air, 80% of the initial efficiency remained, even after an illumination time close to 4000 h in a sealed environment.

Other approaches to achieve 2-D/3-D heterostructures have also been extensively applied. Zhang et al. used bifunctional 5-aminovaleric acid (Ava, NH_2_C_4_H_9_COOH) in MAPbBr_3_ perovskite precursor solutions, and obtained a cross-linked 2-D/3-D Ava(MAPbBr_3_)_n_ structure as confirmed by Raman spectra [164]. They also reported the high phase stability of α-CsPbI_3_ by introducing ethylenediamine cations (EDA^2+^) [30]. The devices based on CsPbI_3_·0.025EDAPbI_4_ perovskite films showed a *PCE* of 11.86%, with a *V_oc_* of 1.15 V, a *FF* of 0.71, and a *J_sc_* of 14.53 mA·cm^−2^. The 2-D EDAPbI_4_ not only disperses into the 3-D CsPbI_3_ film to form a 2-D/3-D heterostructure, it also acts as a blocking layer. Low-toxicity Bi-based 2-D perovskites with small cations have also been adopted to form 2-D/3-D structures. Hu et al. used the addition of highly air-stable MA_3_Bi_2_I_9_ to form a special structure of 2-D platelets/3-D perovskite films [165]. In these films, 2-D MA_3_Bi_2_I_9_ has been observed to be uniformly distributed among 3-D MAPbI_3_ grains. It not only inhibits the non-radiative recombination of charges, but also greatly improves the tolerance of the film to air moisture. After optimization, these PSCs with optimal MA_3_Bi_2_I_9_ content showed a *PCE* of up to 18.97%.

Considering the health and environmental hazards posed by Pb, the development of non-lead PSCs is of great importance. However, in the case of Sn-based perovskites, it is more difficult to control the crystallization properties, film morphology, and defects. Defects are an important factor that can lead to the failure of the device; therefore, developing methods of preparing high-quality and low-defect non-lead perovskite (tin-based) films is critical. Ran et al. developed a bilateral interfacial method to fabricate efficient Sn-based PSCs (Pb-free) by steaming and spin-coating [166]. Using this method, high-quality 2-D/3-D non-lead perovskite films and high-efficiency and high-stability PSCs were realized. *PCE* of up to 6.98% were observed. Shao et al. reported a new record PCE of 9% for Sn-based PSCs [27]. Highly crystalline films were obtained from 0.08-M layered (2-D) Sn perovskite and 0.92-M (3-D) FASnI_3_ solutions. The high *PCE* results from the reduction in trap-assisted recombination events, the low shunt loss of the charge carriers, and the efficient charge collection. Therefore, a hybrid 2-D/3-D tin perovskite is a good way to overcome the bottleneck in tin perovskite PSCs. Sn-based PSCs provide a great opportunity to change the industry and make a significant contribution to future clean and sustainable energy sources by designing solar cells with a perovskite material that is more capable of maintaining thermal electrons. 

In perovskites, the metal halide octahedra are connected in a one-dimensional chain [167,168]. By adjusting the composition of the metals, halides, and organic cations, various combinations can yield different structures [169,170]. Unlike the 2-D materials that have been extensively studied, the investigation of combinations of 1-D and 3-D perovskites has just begun. The application of 1-D/3-D perovskites was first reported by Fan et al. [29]. In this work, 2-(1H-pyrazol-1-yl)pyridine (PZPY) was introduced into lead halide 3-D perovskites, and a 1-D/3-D composite perovskite structure was obtained by in situ growth. The formation of a 1-D perovskite chain [PbX_6_]^4−^ (X is I or Br) is due to its thermodynamic stability and structural flexibility. In consequence, the introduction of 1-D perovskite materials may even block the negative transfer channel formed by A site ions in 3-D perovskites. This suppresses the irreversible degradation of the perovskite, which is mainly the result of the large-scale migration of A site ions, and enables the thermodynamic self-healing of the device. The 1-D/3-D PSCs exhibited a high *PCE* of 18.10%, a *J_sc_* of 21.70 mA cm^−2^, a *V_oc_* of 1.08 V, and an *FF* of 0.77. After aging in an environment with 55% relative humidity at 85 °C for 15 h, the efficiency of the PSCs could be restored to 95% of the initial efficiency with a 25 min recovery process. After five such aging cycles, the retention rate of the *PCE* was over 90%. Thus, the stability of these PSCs was improved significantly, as compared with traditional 3-D PSCs.

In summary, the construction of unique heterogeneous perovskite films by hybridization with different low-dimensional materials is a feasible way to prepare high-efficiency and stable perovskite solar cells. The mixed dimensions can be stacked in different degrees due to the various dimensions of the perovskite layer. This reduces the band gap, which is enlarged by the introduction of organic amine ions. The electron and hole defect densities of the film are reduced by the heterogeneous interface of the perovskite. At the same time, the increase of the depletion region accelerates the transmission of charge carriers and reduces carrier recombination. Extensive studies have demonstrated that the thermal stability of PSCs can be effectively improved, providing new ideas and methods for the preparation of highly efficient and stable PSCs, which can help to promote the industrialization of the technology.

### 5.2. Graded-Dimension Perovskite-Based PSCs

As mentioned above, 2-D structured perovskite materials usually contain large alkyl ammonium cations (such as PEA ^+^, EDA, etc.). However, these large organic groups can easily act as impurities in the film to affect electron transport and increase electron recombination, thus ultimately affecting the photovoltaic performance of the device. Another way to create 2-D structures is to deposit a thin layer on the top of a 3-D perovskite. The results of selected studies are summarized below (as well as in Table 5).

Bai et al. adopted a method similar to in situ growth. An appropriate amount of phenylethylamine in toluene is dissolved in the precursor solution, and spin-coated to form 3-D/2-D graded MAPbI_3_ perovskite films [173]. The 3-D perovskite is first crystallized on the substrate, and then the 2-D perovskite is induced to crystallize on top, forming an ultrathin film. It was observed that the PEI solution in toluene did not affect the quality of perovskite crystallization; instead, the film grains became larger, and defects were cleared away. Yang et al. performed density functional theory (DFT) calculations to understand the role of the hydrophobic layer (tetramethylammonium (TMA), tetraethylammonium (TEA)) [174], and found that the surface functionalization technique incorporated hydrophobic molecules as water-resisting layers on the surface of the perovskite by blocking hydrogen and ionic bonds. Their devices showed almost no loss in *PCE* when kept in a high relative humidity of 90 ± 5% for more than 30 days. Ma et al. proposed CA_2_PbI_4_/MAPbI*_x_*Cl_3−*x*_ perovskite hybrids [31]. These films also act as light absorbers for PSCs. In this way, the CA_2_PbI_4_/MAPbI*_x_*Cl_3−*x*_ hybrid perovskite PSC (*PCE*: 13.86%, *J_sc_* = 19.29 mA·cm^−2^, *V_oc_* = 0.92 V, *FF* = 0.77) was found to be comparable to the 3-D perovskite (*PCE*: 13.12%, *J_sc_* = 18.50 mA·cm^−2^, *V_oc_* = 0.92 V, *FF* = 0.76) at the same conditions. After 220 h, 54% of the initial *PCE* (CA_2_PbI_4_/MAPbI*_x_*Cl_3−*x*_) remained, while the 3-D perovskite device lost all its efficiency within only 50 h. After 40 days, the CA_2_PbI_4_/MAPbI*_x_*Cl_3−*x*_ film still had not degraded. Chen et al. eliminated excess PbI_2_ by spin-coating a 5-AvaI solution onto a perovskite (FAPbI_3_)_0.88_(CsPbBr_3_)_0.12_ film with HI, and significantly reduced the hysteresis [175]. This was attributed to the improved interfacial charge extraction. After 63 days of moisture exposure (~10% humidity), the device retained 98% of its initial *PCE*. Chen et al. used a PEA_2_PbI_4_ capping layer on top of a Cs_0.05_(FA_0.83_MA_0.17_)_0.95_Pb(I_0.83_Br_0.17_)_3_ perovskite film, and the best device that was made in this way showed a *V_oc_* of 1.11 V, a *FF* of 0.73, and a *J_sc_* of 22.89 mA·cm^−2^ [176]. The reason for the high *V_oc_* exhibited by these devices is the induced larger Fermi-level splitting, reduced non-radiative recombination, and optimized energy band alignment. Lin et al. reported 2-D/3-D stacking structures by the reaction of a 3-D perovskite with n-butylamine (BA), resulting in enhanced device efficiency due to passivation and self-healing effects. Cho et al. improved the stability and efficiency of PSCs by growing low-dimensional (2-D) PEA_2_PbI_4_ films on top of 3-D perovskites [177]. The wide band gap of PEA_2_PbI_4_ can block the transfer of excited electrons to the HTM layer. The film also reduced the charge recombination, improving the efficiency of the device. 

Inorganic perovskite materials tend to have a large band gap and narrow absorption range, which limit their photoelectric conversion efficiency. At the same time, in the traditional perovskite cell structure, due to the heavy doping effect of the electron transport layer titanium oxide, the p-n junction in the device is close to the interface between the perovskite and the HTL, leading to the recombination of holes in the transmission process. Zhang et al. formed 0-D/2-D/3-D heterogeneous hybrid perovskites by spin-coating perovskite quantum dots on top of 2-D/3-D structures, greatly enhancing the hole mobility. These PSCs achieved a *V_oc_* as high as 1.19 V, a *J_sc_* of 12.93 mA·cm^−2^, an *FF* of 80.5%, and a *PCE* of 12.39%. This is currently recognized as the highest reported efficiency for CsPbBrI_2_ perovskite solar cells [178]. Such gradient dimension materials can effectively increase the electric field in the system, and improve the hole transmission rate.

Two-dimensional (2-D) perovskite layers using smaller organic cations (FA or MA) have also been attempted. Lee et al. introduced a thin MAPbI_3_ layer on the top of FAPbI_3_ layers to increase the utilization of light at long wavelengths. An average *PCE* of 15.56% was obtained in 2014 [179]. Taek et al. achieved a high efficiency of 21.3% by spin-coating FABr on the top of a (FAPbI_3_)_0.85_(MAPbBr_3_)_0.15_ film, where a non-stoichiometric ratio of FAPbBr_3−*x*_I*_x_* acted as a passivation layer to reduce charge carrier recombination, and as an electron-blocking layer to improve the *V_oc_* without a conspicuous drop in the *J_sc_* [180].

In short, the introduction of a low-dimensional structure changes the interface energy level and weakens the charge recombination between the perovskite and the ETL. In addition, humidity resistance and thermal stability are improved, resulting in a higher *PCE*. It has been found that layered hybrid structures can effectively increase the internal electric field and increase the hole transmission rate. At the same time, the hydrophobicity of perovskite films is increased after modification by low-dimensional materials, also leading to enhanced device stability.

## 6. Other Hybrid PSCs

Each layer in a hybrid PSC has a significant impact on the performance of the device. In the perovskite layer, most of the defects exist at the grains’ boundaries, where carrier recombination and material degradation often occur. Consequently, the stability and efficiency of the device are seriously limited. In order to improve the efficiency and stability of PSCs, the size of the perovskite particles need to be enlarged, or the surface needs to be passivated.

A mesoporous structure is also a kind of hybrid-structured perovskite system. Since perovskite materials have good electron transport properties, large band-gap oxides such as Al_2_O_3_ and ZrO_2_ can also be used to make a porous layer in a perovskite solar cell. Mesoporous TiO_2_ has a large specific surface area, allowing for the maximum adsorption of perovskite materials, and providing a space for the perovskite film to be oriented [98,100,181]. In addition, mesoporous TiO_2_ can be fully exposed to perovskite materials to ensure the maximum amount of photo-induced charge separation and injection. Some oxide nanorods can be used to increase the specific surface area [35,182]. This indicates that oxide nanorods are an effective charge collection system in PSCs.

It has been found that the addition of polymer materials in the precursor solution can improve the morphology and crystallinity of perovskite films. At the same time, the long chain of a polymer can form a mesh that insulates the perovskite from water and oxygen (as seen in Table 6) [36,183,184,185,186,187,188]. Zhao et al. designed a new structure of PSCs, introducing the long-chain hygroscopic polyethylene glycol (PEG) molecule into the perovskite layer as a polymer skeleton by spin-coating. The film quality of the perovskite showed a significant improvement. The efficiency and reproducibility of the resulting PSCs were significantly better, and the maximum efficiency reached 16%. Moreover, these perovskite films could be prepared at 100 °C by a simple and easy method [184]. PCBM has been observed to have a better impact than PEG. Huang et al. used PCBM as a receptor molecule to fill the voids and grain boundaries of perovskite films. In this way, particles in the thin-film device are larger, reducing the grain boundaries and subsequently achieving a higher conversion efficiency and higher fill factor [185]. From another perspective, Zhang et al. noted that the introduction of α-bis-PCBM could reduce water intrusion, thus preventing erosion of the interface. It also reduced the gap of and/or pinholes in the HTL, increased the efficiency of electron extraction, promoted the crystallization of the perovskite, and improved the stability of the PSCs [183]. This promising approach provides a simple route for the manufacture of highly efficient, stable, heterogeneous PSCs. However, there are still issues: is there really significant charge transfer at the perovskite/polymer interfaces? Is it really beneficial for device performance? In 2018, Jiang et al. systematically investigated polymer hybrid PSCs [189]. In this study, a variety of polymers were used in the hybrid perovskites. It was found that all polymers can effectively passivate perovskites, independent of the band gap of the polymer. Using polymer-doped devices can increase the efficiency from 17.43% to 19.19%, while the device stability was also improved.

Hybrids combining nanostructures, such as carbon nanoparticles, graphene oxide, and graphene [34,190,191], with perovskites have also achieved some interesting results. With the addition of nanostructured materials, the resulting perovskite films contain larger grains due to the slowing down of the crystallization process. This leads to better charge mobility and separation, as well as improved thermal stability. However, too much of the hybrid component may destroy the structure of the perovskite and affect its light absorption. Due to strong optical absorption, superfast charge transfer, and high near-infrared response, these hybrid perovskites have potential applications in photodetectors, optical transistors, and other devices [192,193].

## 7. Conclusions

In order to solve the stability issue of PSCs, the best approach is from the perspective of material design. Based on the tolerance factor, a more suitable hybrid combination should be selected to make the final structure of the perovskite lattice more stable, thus improving the stability of the PSCs. By hybridizing various organic cations, metal cations, or halogen anions in an ABX_3_ compound, the band gap and absorption spectra can be adjusted. The optimization of the crystallinity, morphology, and interface in a PSC can improve carrier mobility, reduce series resistance, and greatly improve the photoelectric current.

As mentioned above, the hybridization of perovskites using Cs^+^ cations, metal halides, low-dimensional perovskites, and polymers has driven rapid progress in PSCs. These hybrid perovskite devices show not only significantly increased *PCE* but also substantially improved stability, due to the favorable energy level alignment with the ETL and HTL. Hybridization can also reduce the trap state density, promote passivation effects, and eliminate the hysteresis in the J-V response, in addition to improving stability. This approach has brought PSCs one step closer to industrialization. However, before mass producing real and usable devices, a thorough understanding of the mechanisms governing hybridized perovskite films is still needed.

## Figures and Tables

**Table 1:** Summary of perovskite solar cell performances employing Cs–MA cations. FTO: fluorine-doped tin oxide; ITO: indium oxide; PCBM: phenyl-C_61_-butyric acid methyl ester; *PCE*: power conversion efficiency; PEDOT:PSS: poly(3,4-ethylenedioxythiophene) polystyrene sulfonate; Spiro-OMeTAD: 2,2′,7,7′-tetrakis-(*N*,*N*-di-p-methoxyphenylamine)9,9′-spirobifluorene.

Device Structure	Perovskite	*J_sc_* (mA·cm^−2^)	*V_oc_* (V)	*FF* (%)	*PCE* (%)	Year	Ref.
ITO/PEDOT:PSS/perovskie/PCBM/Al	Cs_0.1_MA_0.9_PbI_3_	10.10	1.05	0.73	7.68	2014	[113]
FTO/c-TiO_2_/mp-TiO_2_/perovskite/Spiro-OMeTAD/Ag	Cs*_x_*MA_1−*x*_PbI_3_	/	/	/	/	2016	[114]
FTO/c-TiO_2_/mp-TiO_2_/perovskite/Spiro-OMeTAD/Ag	Cs_0.1_MA_0.9_PbI_3_	20.97	1.10	0.74	17.08	2017	[118]
FTO/c-TiO_2_/mp-TiO_2_/perovskite/Spiro-OMeTAD/Au	Cs_0.09_MA_0.91_PbI_3_	22.57	1.06	0.76	18.1	2017	[115]
FTO/c-TiO_2_/mp-TiO_2_/perovskite/Spiro-OMeTAD/Au	Cs_0.1_MA_0.9_PbI_3_	19.20	0.96	0.70	13.0	2018	[119]
FTO/c-TiO_2_/mp-TiO_2_/perovskite/Spiro-OMeTAD/Ag	(CsBr)_0.1_ (MAPbI3)_0.9_	22.60	0.93	0.65	13.6	2017	[117]
FTO/c-TiO_2_/mp-TiO_2_/perovskite/Spiro-OMeTAD/Au	(MAPbI3)_0.9_ (CsPbBr3)_0.1_,	22.8	1.05	0.73	17.6	2016	[116]

**Table 2:** Summary of perovskite solar cell performances employing Cs-FA cations.

Device Structure	Perovskite	*J_sc_* (mA·cm^−2^)	*V_oc_* (V)	FF (%)	*PCE* (%)	Year	Ref.
FTO/c-TiO_2_/perovskite/Spiro-OMeTAD/Ag	Cs_0.1_FA_0.9_PbI_3_	23.5	1.06	0.66	16.5	2015	[127]
FTO/c-TiO_2_/mp-TiO_2_/perovskite/Spiro-OMeTAD/Au	Cs_0.2_FA_0.8_PbI_3_	21.5	1.01	0.7	15.69	2016	[128]
FTO/c-TiO_2_/mTiO_2_/perovskite/Spiro-OMeTAD/Au	Cs_0.2_FA_0.8_PbI_2.84_Br_0.16_	21.9	1.07	0.74	17.35	2016	[128]
FTO/NiO/perovskite/PCBM/PFN-Br/Ag	Cs_0.2_FA_0.8_PbI_3_	19.85	1.01	0.71	15.38	2017	[121]
FTO/SnO_2_/C_60_-SAM/perovskite/Spiro-OMeTAD/Au	Cs_0.2_FA_0.8_PbI_3_	21.85	1.06	0.76	17.61	2016	[129]
FTO/SnO_2_/C_60_-SAM/perovskite/Spiro-OMeTAD/Au	Cs_0.2_FA_0.8_PbI_3_ & 0.6% Pb(SCN)_2_	22.25	1.09	0.81	19.57	2016	[129]
ITO/c-TiO_2_/perovskite/Spiro-OMeTAD/Au	FA_0.83_Cs_0.17_Pb(I_0.6_Br_0.4_)_3_	18.34	1.23	0.79	17.8	2018	[130]
FTO/SnO_2_/PCBM/perovskite/Spiro-OMeTAD/Ag	FA_0.83_Cs_0.17_Pb(I_0.6_Br_0.4_)_3_	19.4	1.19	0.78	17.9	2016	[131]
FTO/c-TiO_2_/perovskite/Spiro-OMeTAD/Ag	Cs_0.15_FA_0.85_PbI_3_	22.85	0.91	0.69	14.46	2017	[132]
ITO/SnO_2_/C_60_/perovskite/Spiro-OMeTAD/Ag	FA_0.85_Cs_0.15_Pb(I_0.95_Br_0.05_)_3_	22.4	1.01	0.72	16.2	2016	[133]
ITO/TPD/perovskite/C_60_/Ag	Cs_0.15_FA_0.85_PbI_3_	23.36	1.00	0.64	15.18	2018	[122]
FTO/c-TiO_2_/perovskite/Spiro-OMeTAD/Ag	FA_0.85_Cs_0.15_PbI_3_	21.50	1.08	0.75	17.3	2016	[41]
FTO/SnO_2_/C_60_/perovskite/spiro-OMeTAD/Au	(HC(NH_2_)_2_)_0.83_Cs_0.17_Pb(I_0.6_Br_0.4_)_3_	23.0	1.06	0.75	18.3	2017	[134]
FTO/c-TiO_2_/perovskite/Spiro-OMeTAD/Au	Cs_0.07_FA_0.93_PbI_3_	21.9	0.98	0.72	15.3	2018	[126]

**Table 3:** Performance summary of perovskite solar cells employing multiple cations.

Device Structure	Perovskite	*J_sc_* (mA·cm^−2^)	*V_oc_* (V)	*FF* (%)	*PCE* (%)	Year	Ref.
FTO/c-TiO_2_/mp-TiO_2_/perovskite/PTAA/Au	MA-FAPb-Br-I	25.00	1.10	0.80	22.1	2017	[5]
FTO/SnO_2_/C_60_/perovskite/Spiro-OMeTAD/Ag	(FA_0.83_MA_0.17_)_0.95_ Cs_0.05_Pb(I_0.9_Br_0.1_)_3_	22.6	1.08	0.74	18.0	2016	[133]
ITO/NiO_x_/F_6_TCNNQ/perovskite/PCBM/Zracc/Ag	Cs-FA-MAPb-Br-I	23.18	1.12	0.80	20.86	2018	[70]
FTO/SnO_2_/perovskite/Spiro-OMeTAD/Au	Cs_0.056_FA_0.76_MA_0.15_PbI_2.42_Br_0.48_	22.03	1.15	0.77	19.56	2018	[21]
ITO/PEN/QD-SnO_2_/perovskite/Spiro-OMeTAD/Au	Cs_0.05_(MA_0.17_FA_0.83_)_0.95_Pb(I_0.83_Br_0.17_)_3_	23.05	1.13	0.79	20.79	2018	[146]
ITO/SnO_2_/Perovskite/Spiro-OMeTAD/Ag	FA_0.945_MA_0.025_Cs_0.03_ Pb(I_0.975_Br_0.025_)_3_	24.94	1.12	0.73	20.51	2018	[147]
FTO/QD-SnO_2_/perovskite/PBDBT/Spiro-OMeTAD/Au	(CsPbI_3_)_0.04_(FAPb I_3_)_0.82_(MAPbBr_3_)_0.14_	22.39	1.12	0.79	19.85	2018	[37]
FTO/c-TiO_2_/perovskite/Spiro-OMeTAD/Au	Cs-FA-MAPb-Br-I	21.77	1.13	0.76	18.76	2018	[141]
FTO/c-TiO_2_/mp-TiO_2_/perovskite/Spiro-OMeTAD/Au	Cs_0.06_FA_0.79_MA_0.15_ Pb(I_0.85_Br_0.15_)_3_ & KI	23.2	1.17	0.79	21.5	2018	[22]
ITO/PTAA/perovskite/C_60_/BCP/Cu	MA_0.6_FA_0.38_Cs_0.02_PbI_2.975_Br_0.025_ & 5 mol% MACl	22.70	1.10	0.73	19.2	2017	[59]
ITO/PEDOT:PSS/perovskite/PC_61_BM/Bphen/Al	FA_0.7_MA_0.2_Cs_0.1_Pb (I_5_/_6_Br_1/6_)_3_ & Pb(SCN)_2_	18.21	1.06	0.73	16.09	2017	[148]
FTO/c-TiO_2_/mp-TiO_2_/perovskite/PTAA/Au	(FA_0.79_MA_0.16_Cs_0.05_) Pb(I_0.83_Br_0.17_)_3_	21.5	1.15	0.73	18.10	2017	[149]
FTO/c-TiO_2_/mp-TiO_2_/perovskite/Spiro-OMeTAD/Au	Cs_0.05_(MA_0.15_FA_0.85_)_0.95_ Pb(I_0.85_Br_0.15_)_3_	21.7	1.05	0.75	17.1	2017	[150]
ITO/ZnO/perovskite/Spiro-OMeTAD/Ag	Cs_6_(MA_0.17_FA_0.83_)_94_Pb(I_0.83_Br_0.17_)_3_	22.5	1.12	0.73	18.6	2017	[151]
FTO/bl-TiO_2_/Li-mp-TiO_2_/perovskite/Spiro-OMeTAD/Au	(FAPbI_3_)_0.83_(MAPbBr_3_)_0.17_	23.50	1.07	0.74	21.1	2016	[144]
FTO/c-TiO_2_/Li-mp-TiO_2_/perovskite/Spiro-OMeTAD/Au	Rb_5_(Cs_5_MAFA)_0.95_Pb(IBr)_x_	22.8	1.18	0.81	21.8	2016	[6]
FTO/c-TiO_2_/perovskite/Spiro-OMeTAD/Au	Rb_0.05_Cs_0.05_[(FA_0.83_MA_0.17_)]_0.9_ Pb(I_0.83_Br_0.17)3_	20.65	1.47	0.72	17.02	2018	[145]
FTO/c-TiO_2_/mp-TiO_2_/perovskite/Spiro-OMeTAD/Au	FA_0.80_MA_0.15_Rb_0.05_ PbI_2.55_Br_0.45_	23.2	1.17	0.73	20.0	2017	[152]
FTO/c-TiO_2_/mp-TiO_2_/perovskite/PTAA/Au	Rb-MA-FA-Pb-I-Br	23.24	1.12	0.72	18.80	2016	[153]

**Table 4:** Performance summary of one-dimensional (1-D), two-dimensional (2-D), and three-dimensional (3-D) perovskite solar cells.

Device Sintructure	Perovskite	*J_sc_* (mA·cm^−2^)	*V_oc_* (V)	*FF* (%)	*PCE* (%)	Year	Ref.
ITO/PEDOT:PSS/perovskite/PCBM/Al	CH_3_NH_3_PbI_3_/A_10_C_60_	19.41	0.88	0.82	13.97	2015	[162]
FTO/SnO_2_/PC_61_BM/perovskite/Spiro-OMeTAD/Au	BA_0.05_(FA_0.83_ Cs_0.17_)_0.95_Pb(I_0.8_Br_0.2_)_3_	22.7	1.14	0.8	20.6	2017	[163]
FTO/c-TiO_2_/perovskite/Spiro-OMeTAD /Au	CsPbI_3_•0.025EDAPbI_4_	14.53	1.15	0.71	11.86	2017	[164]
FTO/bl-TiO_2_/perovskite/Spiro-OMeTAD /Au	MAPbI_3_/MA_3_Bi_2_I_9_	23.03	1.09	0.76	18.97	2018	[165]
ITO/PEDOT:PSS/perovskite/C_60_/BCP/Al	FASnI_3_/PEAI	24.1	0.52	0.71	9.01	2018	[27]
FTO/c-TiO_2_/mp-TiO_2_/perovskite/TBP/Au	Cs_0.04_MA_0.16_FA_0.8_PbI_0.85_Br_0.15_/PZPY	21.70	1.08	0.77	18.10	2018	[29]
FTO/c-TiO_2_/mp-TiO_2_/perovskite/Spiro-OMeTAD/Au	CH_3_NH_3_PbI_3_/(HOOC(CH_2_)_4_ NH_3_)_2_PbI_4_	18.84	1.02	0.75	14.6	2017	[161]
FTO/c-TiO_2_/mp-TiO_2_/perovskite/ZrO_2_/Carbon	CH_3_NH_3_PbI_3_/(HOOC(CH_2_)_4_ NH_3_)_2_PbI_4_	23.99	0.85	0.63	12.71	2017	[161]
FTO/c-TiO_2_/mp-TiO_2_/perovskite/Spiro-OMeTAD/Ag	(MAPbI_3_)_1−x_/ [(PEI)_2_PbI_4_]_x_	20.10	1.07	0.73	15.60	2015	[171]
ITO/PEDOT:PSS/Perovskite/PCBM/LiF/Ag	[(PEI)_2_PbI_4_]_x_/ (MAPbI_3_)_1−x_	19.95	1.07	0.72	25.27	2015	[172]
FTO/c-TiO_2_/mp-TiO_2_/perovskite/Spiro-OMeTAD/Au	(IC_2_H_4_NH_3_)_2_ (CH_3_NH_3_)_n−1_Pb_n_I_3n+1_:CH_3_NH_3_PbI_3_	14.88	0.83	0.69	9.03	2016	[160]
ITO/PEDOT:PSS/Perovskite/C60/BCP/Ag	(PEA,FA)SnI_3_	20.07	0.47	0.74	6.98	2018	[166]

**Table 5:** Performance summary of graded hybrid perovskite solar cells.

Device Sintructure	Perovskite	*J_sc_* (mA cm^−2^)	*V_oc_* (V)	*FF* (%)	*PCE* (%)	Year	Ref.
ITO/PEDOT:PSS/perovskite/PCBM/Rhodamine 101/LiF/Ag	CA_2_PbI_4_/MAPbI*_x_*Cl_3−*x*_	19.29	0.92	0.77	13.86	2016	[31]
FTO/TiO_2_/perovskite/Spiro-OMeTAD/Au	CH_3_NH_3_PbI_3_ (TMA)	20.10	0.99	0.65	13.00	2016	[174]
FTO/c-TiO_2_/perovskite/Spiro-OMeTAD/Au	CH_3_NH_3_PbI_3_ (TEA)	19.60	0.99	0.67	12.89	2016	[174]
FTO/c-TiO_2_/mp-TiO_2_/perovskite/CuSCN/Au	(5AVA)_2_PbI_4_/(FAPbI_3_)_0.88_(Cs PbBr_3_)_0.12_ & HI	21.93	1.07	0.72	16.75	2018	[175]
FTO/c-TiO_2_/mp-TiO_2_/perovskite/Spiro-OMeTAD/Au	Cs_0.05_(FA_0.83_ MA_0.17_)_0.95_Pb(I_0.83_Br_0.17_)_3_	22.89	1.11	0.73	18.51	2018	[176]
ITO/PTAA/perovskite/PCBM/C_60_/BCP/Cu	MAPbI_3_/BA	22.49	1.11	0.78	19.56	2018	[28]
ITO/PTAA/perovskite/PCBM/C_60_/BCP/Cu	MAPbI_3_/BAI	22.59	1.09	0.77	18.85	2018	[28]
FTO/c-TiO_2_/mp-TiO_2_/perovskite/Spiro-OMeTAD/Au	FAPbI_3_/MAPbI_3_	20.97	1.03	0.74	16.01	2014	[179]
FTO/c-TiO_2_/mp-TiO_2_/perovskite/Spiro-OMeTAD/Au	(FAPbI_3_)_0.85_(MAPbBr_3_)_0.15_/FAPbBr_3−*x*_I*_x_*	23.18	1.16	0.79	21.31	2017	[180]
FTO/c-TiO_2_/mp-TiO_2_/perovskite/Spiro-OMeTAD/Au	Cs_0.1_FA_0.74_MA_0.13_PbI_2.48_Br_0.39_/PEIA	22.73	1.14	0.76	20.08	2018	[177]
FTO/c-TiO_2_/mp-TiO_2_/perovskite/PTAA/Au	3-D/2-D/0-D CsPbBrI_2_	12.93	1.19	0.80	12.39	2018	[178]
FTO/NiO*x*/perovskite/PCBM/Ag	MAPbI_3_/PEAI	21.8	1.17	0.78	19.89	2017	[173]

**Table 6:** Summary of hybrid composite perovskite/material-based solar cells (PSCs).

Device Structure	Perovskite	*J_sc_*(mA cm^−2^)	*V_oc_* (V)	*FF* (%)	*PCE* (%)	Year	Ref.
FTO/c-TiO_2_/mp-Li-TiO_2_/perovskit/Spiro-OMeTAD/Ag	Cs_0.05_(MA_0.17_FA_0.83_)_0.95_ Pb(I_0.83_Br_0.17_)_3_:carbon nanoparticles	22.10	1.16	0.72	18.30	2018	[34]
FTO/c-TiO_2_/P_61_BM/perovskit/P3HT/Mo_3_/Ag	CH_3_NH_3_PbI_3_:PC _61_BM	26.86	0.9	0.53	12.78	2015	[188]
FTO/SnO_2_/perovskite/Spiro-OMeTAD /Au	(CsPbI3)_0.04_(FAPb_I3_) _0.82_(MAPbBr_3_)_0.14_: PBDB-T	22.39	1.11	0.78	19.38	2018	[35]
ITO/Mixing graphene oxide/perovskite/PCBM/Ag	MAPbI_3_:Mixing graphene oxide	20.71	0.96	0.76	15.20	2017	[190]
FTO/c-TiO_2_/mp-TiO_2_/perovskit/Spiro-OMeTAD/Au	FA_0.85_ MA_0.15_ Pb(I_0.85_ Br_0.15_)_3_:reduced graphene oxides	21.80	1.15	0.74	18.73	2016	[191]
ITO/PEDOT:PSS/Perovskite/Ca/PC_71_BM/Al	CH_3_NH_3_PbI_3_:PC_61_BM	20.20	0.97	0.82	16.0	2016	[185]
FTO/c-TiO_2_/mp-TiO_2_/perovskit/Spiro-OMeTAD/Au	CH_3_NH_3_PbI_3_:4-ABPACl	21.98	1.00	0.70	15.38	2015	[187]
FTO/c-TiO_2_/perovskite/Spiro-OMeTAD/Au	MAI:(Pb(Ac)_2_):PCBM	18.0	1.08	0.75	14.4	2015	[186]
FTO/c-TiO_2_/mp-TiO_2_/perovskite/Spiro-OMeTAD/Au	CH_3_NH_3_PbI_3−*x*_Cl*_x_*: C_60_SAM	19.60	0.84	0.72	11.7	2013	[36]
FTO/c-TiO_2_/mp-TiO_2_/perovskite/Spiro-OMeTAD/Au	(FAI)_0.81_(PbI_2_)_0.85_ (MABr)_0.15_(PbBr_2_)_0.15_: α-bis-PCBM	23.73	1.11	0.73	20.80	2017	[183]
FTO/c-TiO_2_/perovskite/Spiro-OMeTAD/Au	CH_3_NH_3_PbI:PC_61_BM	22.5	0.98	0.72	15.4	2016	[184]
FTO/c-TiO2/perovskite/Spiro-OMeTAD/Au	CH_3_NH_3_PbI:J71	22.31	1.11	0.78	19.19	2018	[189]
FTO/c-TiO_2_/perovskite/Spiro-OMeTAD/Au	CH_3_NH_3_PbI:J50	22.28	1.10	0.76	18.81	2018	[189]
FTO/c-TiO2/perovskite/Spiro-OMeTAD/Au	CH_3_NH_3_PbI:J51	22.11	1.10	0.77	18.92	2018	[189]
FTO/c-TiO_2_/perovskite/Spiro-OMeTAD/Au	CH_3_NH_3_PbI:J61	22.35	1.10	0.75	18.69	2018	[189]
FTO/c-TiO_2_/perovskite/Spiro-OMeTAD/Au	CH_3_NH_3_PbI:N2200	22.27	1.10	0.77	19.07	2018	[189]
FTO/c-TiO_2_/perovskite/Spiro-OMeTAD/Au	CH_3_NH_3_PbI:PMMA	21.71	1.11	0.76	18.40	2018	[189]

## Abbreviation

0-D	zero-dimensional
1-D	one-dimensional
2-D	two-dimensional
3-D	three-dimensional
4-ABPACl	butylphosphonic acid 4-ammonium chloride
α-bis-PCBM	α bis (1-[3-(methoxycarbonyl) propyl]-1-phenyl)-[6.6] M
BCP	bathocuproine
BEI	Butylamine iodide
BL	blocking layer
BA	butylamine
Cs	Cesium
CB	conduction band
C_60_SAM	C_60_self-assembled monolayer
CA	cyclopropylamine
CHMA	cyclohexylammonium iodide
DFT	density functional theory
ETL	electron transporting layer
ETM	electron transporting material
EDA	ethylenediamine
FTO	fluorine-doped tin oxide
FA	Formamidinium
FAI	Formamidinium iodide
FF	fill factor
HTM	hole transport material
HTL	hole transporting layer
ITO	indium tin oxide
LUMO	lowest unoccupied molecular orbital
MA	methylammonium
MP	mesoporous
NERL	National renewable energy laboratory
PCBM	[6,6]-phenyl-C61-butyric acid methyl ester
PC_61_BM	[6,6]-phenyl-C_61_-butyric acid methyl ester
PSCs	perovskite solar cells
PCE	power conversion efficiency
PEDOT:PSS	poly(3,4-ethylenedioxythiophene) poly(styrenesulfonate)
PFNBr	poly9,9-bis6-(*N,N,N*-trimethylammonium)hexylfluorene-alt-co-phenylenebromide
PTAA	polytriarylamine
PMMA	Polymethyl methacrylate polymer
PTAA	poly-(3-thiopheneaceticacid)
PEG	polyethylene glycol
PEAI	phenylethylammonium iodide
PZPY	3-(2-pyridyl)-pyrazol-1-yl
PEA	phenylethylammonium
PEI	poly(ethyleneimine)
PC71BM	[6,6]-phenyl C71butyric acid methyl-ester
PBDB-T	poly[(2,6-(4,8-bis(5-(2-ethylhexyl)thiophen-2-yl)benzo[1,2-b:4,5-b′]dithiophene)-co-(1,3-di(5-thiophene-2-yl)-5,7-bis(2-ethylhexyl)benzo[1,2-c:4,5-c′]dithiophene-4,8-dione)]
Pd	Plumbum
Rb	Rubidium
RMS	root mean square
Sn	Stannum
SCN	thiocyanate
spiro-OMeTAD	2,2′,7,7′-tetrakis-(*N*,*N*-di-p-methoxyphenylamine)9,9′-spirobifluorene
TBP	4-*tert*-butylpyridine
TMA	tetramethylammonium
TEA	tetra-ethyl ammonium
VB	valence band
